# Antenna-Biased Odorant Receptor PstrOR17 Mediates Attraction of *Phyllotreta striolata* to (S)-Cis-Verbenol and (−)-Verbenone

**DOI:** 10.3390/ijms25084362

**Published:** 2024-04-15

**Authors:** Zhanyi Xu, Peitong Chen, Ru Yan, Guoxing Chen, Jiali Qian, Guonian Zhu, Mengli Chen, Yirong Guo

**Affiliations:** 1 Key Laboratory of Biology of Crop Pathogens and Insects of Zhejiang Province, Ministry of Agriculture Key Laboratory of Molecular Biology of Crop Pathogens and Insects, Institute of Pesticide and Environmental Toxicology, Zhejiang University, Hangzhou 310058, China; 20156820@zju.edu.cn (Z.X.); 12216136@zju.edu.cn (P.C.); jialiqian@zju.edu.cn (J.Q.); zhugn@zju.edu.cn (G.Z.); 2 College of Life Sciences, Zhejiang University, Hangzhou 310058, China; yanru@zju.edu.cn; 3 Key Lab for Biology of Crop Pathogens and Insect Pests and Their Ecological Regulation of Zhejiang Province, College of Advanced Agricultural Sciences, Zhejiang A&F University, Hangzhou 311300, China; guoxingchen@stu.zafu.edu.cn

**Keywords:** *Phyllotreta striolata*, odorant receptor, (−)-verbenone, (S)-cis-verbenol, attraction, single sensillum recording, molecular docking

## Abstract

*Phyllotreta striolata*, the striped flea beetle, is one of the most destructive pests in Brassicaceae plants worldwide. Given the drawbacks associated with long-term use of chemical insecticides, green strategies based on chemical ecology are an effective alternative for beetle control. However, the lack of information on beetle ecology has hindered the development of effective biocontrol strategies. In this report, we identified two odorants, (S)-cis-verbenol and (−)-verbenone, which displayed significant attraction for *P. striolata* (*p* < 0.05), indicating their great potential for *P. striolata* management. Using the *Drosophila* “empty neuron” system, an antenna-biased odorant receptor, PstrOR17, was identified as responsible for the detection of (−)-verbenone and (S)-cis-verbenol. Furthermore, the interactions between PstrOR17 and (−)-verbenone or (S)-cis-verbenol were predicted via modeling and molecular docking. Finally, we used RNAi to confirm that PstrOR17 is essential for the detection of (−)-verbenone and (S)-cis-verbenol to elicit an attraction effect. Our results not only lay a foundation for the development of new and effective nonchemical insecticide strategies based on (S)-cis-verbenol and (−)-verbenone, but also provide new insight into the molecular basis of odorant recognition in *P. striolata*.

## 1. Introduction

The striped flea beetle, *Phyllotreta striolata* (Fabricius) (Coleoptera: Chrysomelidae), a notorious pest of Brassicaceae crops, is widely distributed worldwide [1]. Adults chew on leaves, while larvae mainly feed on roots underground, resulting in considerable economic loss. In southern China, *P. striolata* occurs in six to nine generations throughout the year without overwintering [2]. With the expansion of vegetable acreage, the geographic range of *P. striolata* and the damage it causes have increased in China and North America, making it one of the most threatening pests of Brassicaceae crops [3,4]. Currently, the management of *P. striolata* primarily relies on chemical insecticides [5,6]. However, long-term overuse of chemical insecticides has caused the rapid development of resistance, environmental pollution, and food safety concerns [7]. Thus, alternative effective strategies for *P. striolata* control are urgently required, and developing pest olfactory behavior regulators based on the sensitive olfactory system is recognized as a promising strategy in an IPM project.

The aggregation pheromone and isothiocyanates (ITCs) from Brassicaceae plants encourage attraction in *P. striolata* [8,9]. Commercial attractants have been developed based on the aggregation pheromone to control *P. striolata* in the field. However, besides the aggregation pheromone and ITCs, no other odorants have been discovered to elicit behavioral responses in *P. striolata*. Odorants (−)-verbenone and (S)-cis-verbenol can be found in various common bark beetle host species, as well as in various angiosperm species such as Brassica and Sinapis, which are host plants of *P. striolata* [10,11]. Behavioral responses to verbenone and verbenol are common among pest bark beetles, and these have shown considerable potential in management [12]. However, the molecular mechanisms underlying the detection of (−)-verbenone and (S)-cis-verbenol remain unclear.

To perceive chemical signals from a complex environment, insects have evolved highly sensitive olfactory systems that are essential for insect behaviors, such as host identification, mating, oviposition, and predator avoidance [13,14,15,16]. Insects detect odorants via odorant receptors (ORs), and the activated ORs convert chemical signals into electrical signals, which are transmitted to the brain via the olfactory sensory neuron (ORN) axon, generating the corresponding behavioral responses [17,18]. OR proteins play a pivotal role in insect odorant detection [19]; thus, investigation of OR functionality is essential for understanding the olfactory recognition mechanism of the insect peripheral nervous system.

Since the discovery of the first insect OR in *Drosophila melanogaster* [20], numerous candidate ORs have been identified in many insect orders, shedding light on the molecular mechanisms of odorant detection in insects [21,22]. Heterologous expression techniques, including *Xenopus oocytes*, human embryonic kidney 293 (HEK293) cells, and the *Drosophila* “empty neuron” system, have been effectively used for the deorphanization of insect ORs [23,24,25,26]. Through these techniques, many candidate ORs have been characterized for detecting plant volatiles, insect pheromones, and ovipositing attractants and repellents. For example, the HarmOR42 orthologous ORs are specifically responsive to phenylacetaldehyde, a floral odor commonly found in angiosperms [27]. In *Plutella xylostella*, OR35 and OR49 are essential for detecting ITCs, governing oviposition and host recognition [13]. Moreover, the olfactory mechanisms underlying sex pheromone communication have been elucidated in *Campoletis chlorideae* and *Microplitis mediator* [28,29,30]. However, besides 15 ORs characterized functionally across a few species, the functions of ORs in the largest insect order, Coleoptera, remain largely unexplored [22,23,31,32,33,34,35].

In a previous study, 73 OR genes were predicted from *P. striolata*, of which 34 ORs were full-length transcripts, encoding proteins with >310 amino acids [36]. However, none of these ORs have been characterized except for the odorant receptor coreceptor (Orco), leaving all ORs as orphans. In this study, we investigated the behavioral and electrophysiological responses of *P. striolata* to (−)-verbenone and (S)-cis-verbenol. To gain insights into the molecular mechanisms underlying the detection of these two odorants in *P. striolata* adults, the individual PstrORs were heterologously expressed using the *Drosophila* “empty neuron” system. Subsequently, molecular docking was performed to explore the interaction of PstrOR and odorants. We finally demonstrated that PstrOR17 was responsible for detection of (−)-verbenone and (S)-cis-verbenol through RNAi and behavioral assays. Our findings provide valuable support for the development of biological pest strategies based on olfactory-active compounds.

## 2. Results

### 2.1. (−)-Verbenone and (S)-Cis-Verbenol Elicit Behavioral and Electrophysiological Responses in P. striolata Adults

**Figure 1:**
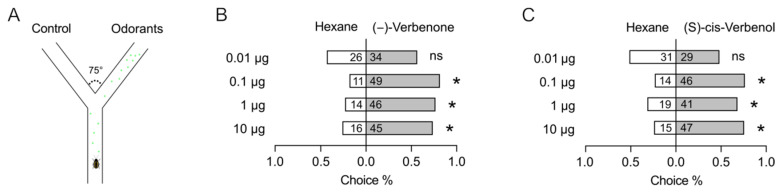
The behavioral responses of *P. striolata* to (−)-verbenone and (S)-cis-verbenol. (**A**) Schematic of the Y-tube olfactometer. (**B**) Behavioral responses of *P. striolata* to (−)-verbenone. (**C**) Behavioral responses of *P. striolata* to (S)-cis-verbenol. Data are presented as mean ± SEM. Numbers in panel B and C represent the replicates. The χ^2^ goodness-of-fit test was used to assess the preferences of *P. striolata* adults between controls and treatments. * Indicates a significant difference (*p* < 0.05), and ns indicates no significant difference.

We used the two-choice assay in a Y-tube olfactometer to examine the behavioral responses of *P. striolata* adults to (−)-verbenone and (S)-cis-verbenol (Figure 1A). Both (−)-verbenone and (S)-cis-verbenol significantly attracted the adults of *P. striolata* to a comparable degree and in a dose-dependent manner (Figure 1B,C).

**Figure 2:**
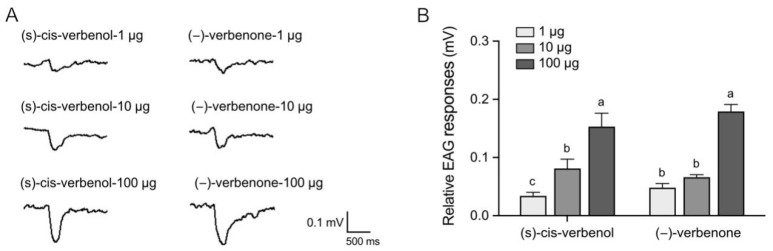
The EAG responses of *P. striolata* to (−)-verbenone and (S)-cis-verbenol. (**A**) Traces of EAG responses of *P. striolata* to different doses of (S)-cis-verbenol and (−)-verbenone. (**B**) EAG responses of *P. striolata* to (S)-cis-verbenol and (−)-verbenone (n = 20). Data are presented as mean ± SEM. Different letters above the bars indicate significant difference among different treatments at the *p* < 0.05 level (one-way ANOVA with Duncan’s post hoc multiple comparisons).

To evaluate whether the attraction elicited by (−)-verbenone and (S)-cis-verbenol was mediated by the olfactory system or not, we examined the electrophysiological responses of the antennae of *P. striolata* adults to two odorants using electroantennogram (EAG) recording. We found both (−)-Verbenone and (S)-cis-verbenol elicited EAG responses, and the responses increased gradually with increasing concentration (Figure 2). These results indicated that (−)-verbenone and (S)-cis-verbenol were perceived by antennae of *P. striolata* adults to elicit attraction behavior, suggesting that the attraction of (−)-verbenone and (S)-cis-verbenol to *P. striolata* adults was probably mediated through the olfactory system. 

### 2.2. PstrOR17 Narrowly Tuned to (−)-Verbenone and (S)-Cis-Verbenol 

**Figure 3:**
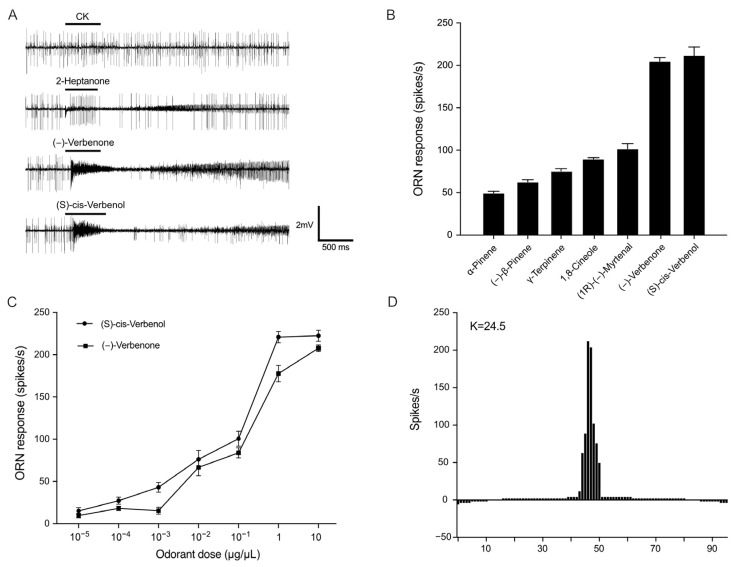
PstrOR17 is activated by (−)-verbenone, (S)-cis-verbenol, and five other structurally related compounds. (**A**) Representative single sensillum recording traces of *Drosophila* ab3A ORNs expressing PstrOR17 to odorants in 10 μg/μL. (**B**) Odorant response profiles of PstrOR17 expressed in *Drosophila* ab3A ORNs (n = 8). Data are presented as mean ± SEM. (**C**) Dose-response curves for PstrOR17 to (−)-verbenone and (S)-cis-verbenol (n = 8). Data are presented as mean ± SEM. (**D**) Tuning curves of PstrOR17. The 96 odorants are displayed along the horizontal axis with those eliciting the strongest response placed near the center and those eliciting the weaker responses placed near the edges. The tuning breadth is represented by the kurtosis values (K) of the distribution. A greater K values represent narrow tuning spectra and smaller ones represent broader spectra.

Since *P. striolata* adults exhibited considerable attraction to odorants (−)-verbenone and (S)-cis-verbenol, we hypothesized PstrORs with high transcripts were primarily responsible for detection of these two odorants. Thus, we cloned several abundant OR genes from *P. striolata* antenna, including *PstrOR9*; *PstrOR11*; *PstrOR17*; *PstrOR26*; *PstrOR38*; and *PstrOR59*, and heterologously expressed individual *PstrORs* in *D. melanogaster* ab3A OSNs lacking endogenous OR. We then examined the responses of the PstrORs-expressing ab3A neurons to (−)-verbenone and (S)-cis-verbenol via single sensillum recording and showed that flies expressing with PstrOR17 conferred a strong response to (−)-verbenone and (S)-cis-verbenol (Figure 3A,B). In addition, concentration gradient assays revealed that PstrOR17 responded to both (−)-verbenone and (S)-cis-verbenol in a dose-dependent manner (Figure 3C). Furthermore, we investigated the responses of PstrOR17 to 94 plant volatiles (Table S1) to identify whether PstrOR17 was tuned to other odorants. It turned out PstrOR17 also conferred moderate responses to five other odorants: α-pinene; (−)-β-pinene; γ-terpinene; 1,8-cineole; and (1R)-(−)-myrtenal (Figure 3B,D). However, PstrOR9, PstrOR11, and PstrOR38 were not functionally expressed in the *D. melanogaster* ab3A OSNs, and flies expressing PstrOR26 or PstrOR59 were not activated by any of the odorants we tested.

### 2.3. PstrOR17 Sequence Analysis

**Figure 4:**
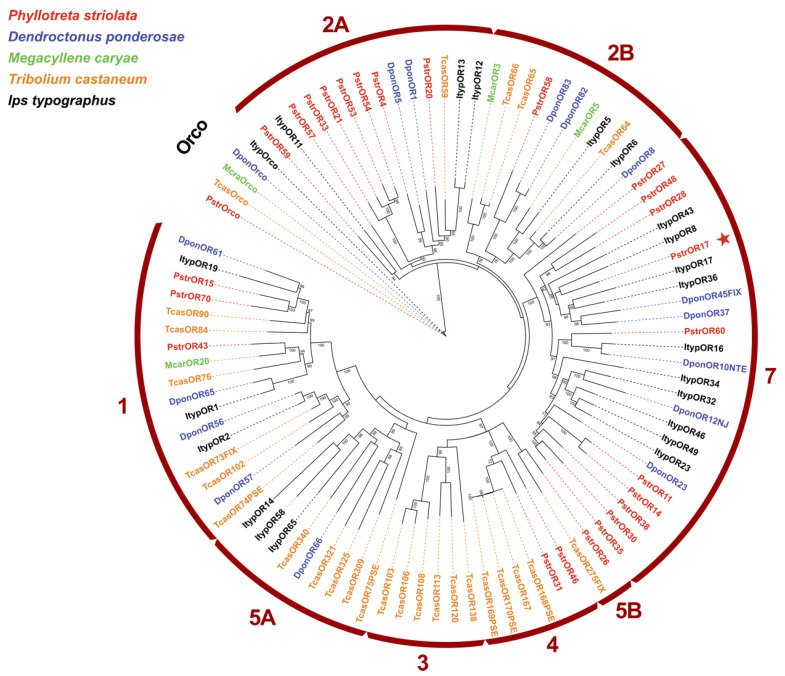
The phylogenetic analysis of PstrOR17. The tree is based on a MAFFT alignment of amino acid sequences constructed using IQ-TREE and rooted with the Orco lineage. The receptor sequences included were from *Phyllotreta striolata* (Pstr, red); *Dendroctonus ponderosae* (Dpon, blue); *Ips typographus* (Ityp, black); *Megacyllene caryae* (Mcar, green); and *Tribolium castaneum* (Tcas, orange). Numbers at nodes represent bootstrap support (n = 1000), calculated using IQ-TREE, and are only shown for main lineages and if ≥70. The major Coleopteran OR subfamilies are indicated with red arcs and numbers. The red star indicates the position of PstrOR17.

The ORF of the *PstrOR17* gene is composed of 1182 bp and encodes a protein of 393 amino acids. A phylogenetic tree was constructed using the candidate ORs from several Coleoptera species including *Dendroctonus ponderosae*, *Megacyllene caryae*, *Tribolium castaneum*, *Ips typographus*, and *P. striolata* (Figure 4). A recent study classified and revised nine high-order monophyletic OR subfamilies (designated groups 1, 2A, 2B, 3, 4, 5A, 5B, 6, and 7) across the Coleoptera [37]. Phylogenetic analysis showed that the largest number of PstrORs was found in group 7 (10 ORs, including PstrOR17); followed by group 2A (8 ORs); group 1 (3 ORs); group 4 (2 ORs); and group 2B (1 OR).

### 2.4. PstrOR17 Was Highly Expressed in Antennae 

**Figure 5:**
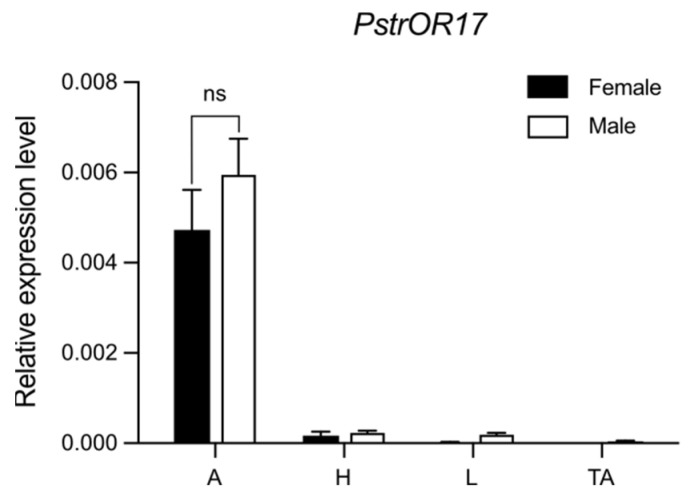
The relative expression level of *PstrOR17* in different tissues. Antennae (A), heads without antennae (H), legs (L), thoraxes and abdomens without legs (TA). Data are presented as mean ± SEM, n = 3. The Student’s t-test was used to evaluate differences in the expression level of *PstrOR17* between sexes; ns indicates no significant difference in the expression level in antennae between sexes.

The expression pattern of *PstrOR17* in different tissues of male and female *P. striolata* adults was evaluated using RT-qPCR. *PstrOR17* was expressed to the highest degree in antennae (A), and with no significant difference in expression between males and females (Figure 5). *PstrOR17* was rarely expressed in other tissues, such as heads without antennae (H), legs (L), and thoraxes and abdomens without legs (TA).

### 2.5. Modeling and Molecular Docking of PstrOR17

**Figure 6:**
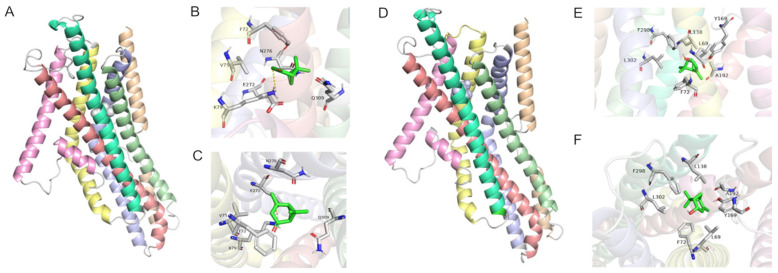
The 3D structure and molecular docking of PstrOR17. (**A**) Binding model of (−)-verbenone with PstrOR17. The helix and loop structure of PstrOR17 is indicated by rainbow colors, and the (−)-verbenone binding conformation is indicated in gray. (**B**,**C**) The front and top view of the main region of PstrOR17 binding with (−)-verbenone. Dashed rosy lines represent hydrogen bonds. (**D**) Binding model of (S)-cis-verbenol with PstrOR17. The helix and loop structure of PstrOR17 is indicated by rainbow colors, and the (S)-cis-verbenol binding conformation is indicated in gray. (**E**,**F**) The front and top view of the main region of PstrOR17 binding with (S)-cis-verbenol.

To understand the interactions of active odorants with PstrOR17, we predicted the 3D structure of PstrOR17 and performed molecular docking simulation. The 3D model of PstrOR17 with helical and loop structures is shown in Figure 6A,D. The reliability of the model structure was evaluated using a PROCHECK Ramachandran plot, indicating that 96.5% of the PstrOR17 residues were placed in the most favored regions (A, B, L) (Figure S1). Molecular docking predicted that (−)-verbenone interacted with Lys 79 via a hydrogen bond of 3.21 Å length (Figure 6B,C). An additional five residues, Phe 72; Val 75; Glu 272; Asn 276; and Gln 309, with the above residue together mediated the interaction between PstrOR17 and (−)-verbenone. Seven residues of PstrOR17 were predicted to form the binding site with (S)-cis-verbenol (Figure 6E,F). Furthermore, Phe 72 of PstrOR17 was the common residue for both ligands. 

### 2.6. Knockdown of PstrOR17 Attenuates Behavioral Responses to (−)-Verbenone and (S)-Cis-Verbenol

**Figure 7:**
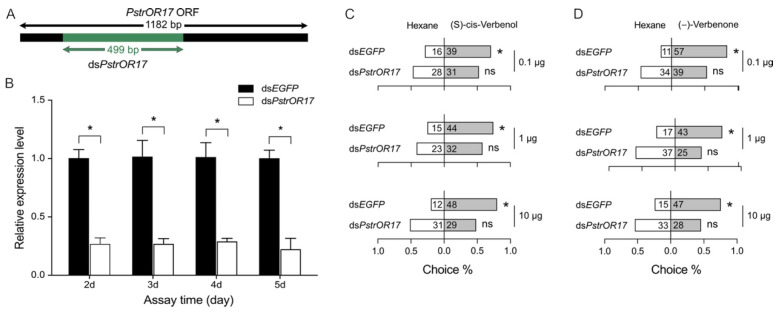
The behavioral response of *P. striolata* to (−)-verbenone and (S)-cis-verbenol after *PstrOR17* knockdown by RNAi. (**A**) Schematic of *PstrOR17* and the region used for dsRNA synthesis. A gene fragment (green) of 499 bp near the 5′-end of *PstrOR17* was used for dsRNA synthesis. (**B**) Relative expression level of *PstrOR17* from days 2–5 after dsRNA treatment. Data are presented as mean ± SEM, n = 3. The Student’s *t*-test was used to evaluate the knockdown efficiency of dsRNA. * Indicates a significant difference (*p* < 0.05). (**C**) Behavioral responses of *P. striolata* to (S)-cis-verbenol after ds*EGFP* or ds*PstrOR17* treatment. (**D**) Behavioral responses of *P. striolata* to (−)-verbenone after ds*EGFP* or ds*PstrOR17* treatment. Data are presented as mean ± SEM. The numbers in panels C and D represent the replicates. The χ^2^ goodness-of-fit test was used to assess the preferences of *P. striolata* adults between controls and treatments. * Indicates a significant difference (*p* < 0.05), and ns indicates no significant difference.

To confirm the role of PstrOR17 in vivo, the expression of *PstrOR17* was knocked down via injection of dsRNA. RT-qPCR results showed that the relative expression level of *PstrOR17* was significantly decreased after dsRNA injection compared with that of the ds*EGFP* treatment (Figure 7B). The knockdown effect of *PstrOR17* was maintained for at least 5 days (Figure 7B). Next, we tested the behavioral responses of *P. striolata* to (−)-verbenone and (S)-cis-verbenol on the 3–5 days after dsRNA treatment. Behavioral assays indicated that the preference of beetles to (S)-cis-verbenol or (−)-verbenone almost disappeared with the knockdown of *PstrOR17* (Figure 7C,D).

## 3. Discussion

Many herbivorous insects rely on olfaction for accurate and efficient host colonization, exhibiting an intrinsic behavioral response to volatiles emitted by hosts or the environment. Insect ORs are the key factors in determining the specificity and sensitivity of olfaction [38]. In recent years, several plant-derived attractants or repellents targeting insect sensitive olfactory systems have demonstrated excellent practical implications in plant protection as alternatives to pesticides. For example, methyl eugenol is widely used as a commercial attractant for *Bactrocera dorsalis* [39]. Furthermore, synthetic pheromones disrupt mating, providing another efficient method for pest management which has been successfully used in numerous moth species [40]. In this study, we reported that *P. striolata* adults were attracted by (S)-cis-verbenol and (−)-verbenone, which contributed to the development of plant-derived attractants for future pest management of *P. striolata*.

Verbenone and verbenol are not only identified as pheromone components of many species of *Dendroctonus*, *Tomicus*, *Pityogenes*, and *Ips* [41], but also released by various gymnosperm tree species [42,43] and angiosperm species [10,11]. Currently, verbenone and verbenol are recognized as “universal bark beetle repellents” due to their effective repellent properties [12]. In addition to working as beetle repellents, verbenone and verbenol also attract other insects. For instance, cis-verbenol acted as a cue to attract solitary egg parasitoid *Anaphes nitens* for host location and selection [44]. Verbenone and cis-verbenol, functioning as pheromones of *Gonipterus platensis*, elicited weevil attraction [45]. Addition of (−)-verbenone to sticky traps significantly increased the catches of western flower thrips *Frankliniella occidentalis* [46]. Our findings reveal a notable preference of *P. striolata* for (S)-cis-verbenol and (−)-verbenone, suggesting their potential to effectively trap and confuse *P. striolata*.

To identify the OR responsible for recognizing (−)-verbenone and (S)-cis-verbenol, we cloned and deorphanized several abundant ORs from both male and female antennae, since both sexes of beetles were attracted to these two odorants. Currently, various approaches are employed to deorphanize ORs in insects, of which *Xenopus* oocytes and HEK 293 cell lines are commonly used for functional characterization studies of ORs across different insect orders [23]. However, a previous study indicated that the *Drosophila* “empty neuron” systems proved to be more accurate to validate the function of a specific OR [26]. Numerous ORs from Diptera and Lepidoptera were functionally characterized through *Drosophila* “empty neuron” systems. In addition, Antony et al. found that transgenic *Drosophila* olfactory neurons expressing *OR1* from *Rhynchophorus ferrugineus* respond to ferrugineol and ferrugineone, indicating *Drosophila* ORNs could be efficiently used to study ORs from Coleoptera [31]. Thus, we also used *Drosophila* “empty neuron” systems to characterize PstrORs and identified that the neuron expressing PstrOR17 was strongly activated either by (−)-verbenone or (S)-cis-verbenol. In addition, PstrOR17 could recognize five other structurally related compounds, including α-pinene; (−)-β-pinene; γ-terpinene; 1,8-cineole; and (1R)-(−)-myrtenal, four of which were related to bark beetle pheromone. A common defensive host monoterpene, α-Pinene, is also a precursor for the bark beetle pheromones (−)-verbenone and (S)-cis-verbenol [41]. γ-Terpinene, (1R)-(−)-myrtenal, and 1,8-cineole were detected and identified in pine volatiles, regarded as the pheromone candidates for the bark beetle [44,45]. Previous studies reported (6R,7S)-himachala-9,11-diene as the aggregation pheromone of *P. striolata* [8,9]; however, our results and the cited literature suggest that whether these identified compounds are pheromone analogs of *P. striolata* remains to be further investigated. 

The phylogenetic analysis revealed that the majority of PstrORs were clustered within group 7, which included PstrOR17. Similarly, RferOR1, pheromone receptor of *R. ferrugineus*, was also found to belong to group 7 [31]. In *I. typographus*, two functionally characterized pheromone receptors, ItypOR46 and ItypOR49, were located in group 7, which contained the largest number of ItypORs [22]. These suggest that PstrOR17 may serve as a potential pheromone receptor for *P. striolata*. However, previous studies reported that the characterized pheromone receptors from other Coleoptera species, such as *D. ponderosae* and *M. caryae*, were distributed in group 1 and group 2B [23,34]. Therefore, the pheromone receptors of Coleopteran species do not cluster in specific clades as they do in Lepidopteran species. Further investigation is necessary to determine whether PstrorOR17 is a candidate pheromone receptor of *P. striolata*.

In recent years, the accuracy of protein structures predicted using artificial intelligence models has been improved with the application of AlphaFold2 and Rosettafold software [47,48]. Moreover, molecular docking has been employed to explore the binding properties of insect ORs and odorants. Previous studies showed that site-directed mutagenesis of key residues predicted via molecular docking decreased the binding affinity of the mutant protein [22,49]. These studies indicated that the combination of model and molecular docking was a feasible technique to predict the interaction of ORs and ligands. Therefore, we used AlphaFold2 modeling and molecular docking to predict the binding site of PstrOR17 to (−)-verbenone and (S)-cis-verbenol. The results indicated that (−)-verbenone and (S)-cis-verbenol interacted with PstrOR17 in the identified active sites. In addition, PstrOR17 and these two ligands had an identical Phe 72 residue, but whether this is the key residue responsible for PstrOR17-ligand interactions needs to be verified through further experiments, such as site-directed mutagenesis.

To verify the function of PstrOR17 in vivo, we employed RNAi-based gene silencing to silence *PstrOR17* expression. Recently, RNAi has been successfully used in functional studies for downregulation of target genes for several insect species, particularly chemosensory genes [28,33,50]. Numerous studies reported that knockdown of ORs consistently produces attenuated behavioral responses. For instance, knockdown of CchlOR18 or CchlOR47, which are tuned to 14:Ald or 2-Hep, respectively, resulted in males losing preference for 14:Ald or 2-Hep in *Campoletis chlorideae* [28]. In our study, when the transcript level of PstrOR17 was reduced, the attractant activity of *P. striolata* induced by (−)-verbenone and (S)-cis-verbenol nearly disappeared. This observation strongly suggests that PstrOR17 was responsible for detecting (−)-verbenone and (S)-cis-verbenol to elicit attraction in *P. striolata*.

## 4. Materials and Methods

### 4.1. Insect Rearing

*P. striolata* adults were collected from the experimental field with cruciferous crops at South China Agricultural University, Guangdong in China (113.3° E, 23.1° N). Collected beetles were reared on pakchoi (*Brassica chinensis* L.) and maintained at 25 ± 1 °C under 60–80% relative humidity (RH) with a photoperiod of 14:10 (L:D).

Flies were reared on standard cornmeal–yeast agar medium (Genesee Scientific, San Diego, CA, USA), and at 25 ± 1 °C under 60% RH with a light/dark photoperiod of 12 h each. 

### 4.2. Compounds

Compounds used in electrophysiological recordings and behavioral assays are listed in Table S1.

### 4.3. Behavioral Assays

The two-choice behavioral assay was performed using a glass Y-tube olfactometer, which consisted of a 12 cm–long main arm and two 10 cm–long side arms with a 75°-angle and a 1.5 cm inner diameter. One piece of filter paper (20 × 30 mm) loaded with 10 μL of compound solution was placed in a 250 mL glass flask as the odor source, connected to one arm, and the other arm served as control, containing a filter paper with the same volume of solvent alone. Tested chemicals were diluted in a concentration series (10^−3^ to 1 mg/mL) in hexane. Moistened, activated charcoal-filtered air was pumped into each arm at a flow rate of 0.2 L/min. During the behavioral assay, a beetle starved for 24 h was placed at the beginning of the main arm. Behavior was recorded as the choice of compound or control if the beetle reached the middle of the side arm within 2 min and remained there for at least 10 s. To avoid a potential side bias, the arms of the Y-tube olfactometer were reversed every five replications. At least 50 replicates were performed per treatment, and each beetle was only used once.

### 4.4. Electroantennogram

The electroantennogram (EAG) procedure was followed as previously described with minor modifications [33]. Briefly, the head was excised and the proximal tip of the antenna was finely transected. Then, the tip of the antenna and the head were attached to the recording electrode and reference electrode, respectively. The antenna preparation was bathed in a high humidity air stream flowing at 20 mL/s and a stimulus pulse was delivered for 0.5 s at 2 mL/s. To avoid sensory adaptation, two stimulations were separated by 30 s intervals. EAG signals were amplified via IDAC-4 (Syntech, Kirchzarten, Germany), and recordings were analyzed with EAG pro 2000 software (Syntech, Kirchzarten, Germany). Each compound was diluted in hexane. For each compound, 10 μL solution was applied on a filter strip (0.2 × 30 mm), which was inserted into a Pasteur pipette to build a stimulus cartridge. Pipettes with filter papers containing hexane alone served as negative control. A series of concentrations (10^−1^ to 10 mg/mL) of compounds were tested. EAG response values were calculated by subtracting the average response elicited by hexane. Twenty repeats were conducted per compound.

### 4.5. RNA Extraction and cDNA Synthesis

The antennae (A, 200 of each sex); heads without antennae (H, 20); legs (L, 50); and thoraxes and abdomens without legs (TA, 10) were collected from male or female adult beetles. Collected tissues were immediately transferred to liquid nitrogen and stored at −80 °C before RNA extraction. Total RNA was extracted using RNAiso Plus (TaKaRa, Dalian, China), following the manufacturer’s instructions. RNA concentration and purity were checked with a spectrophotometer (Allsheng, Hangzhou, China) and 1% agarose electrophoresis. The PrimeScript™ RT reagent Kit with gDNA Eraser (TaKaRa, Dalian, China) was used to synthesize cDNA, following the manufacturer’s instructions.

### 4.6. Generation of UAS Line Constructs

Transgenic fly strains were generated as described previously [25]. The ORF of *PstrORs* was individually amplified from antennal cDNA using specific primers (Table S2), and cloned into a *pUAST.attb* vector using the pEASY-Basic Seamless Cloning and Assembly Kit (TransGen Biotech, Beijing, China). The recombinant *pUAST.attb-PstrOR* plasmid was injected into *M{3xP3-RFP.attP}ZH-86Fb* embryos by UniHuaii Co., Ltd. for PhiC31 integration. Transformants were selected from individually injected flies with the compound eye-color rescue phenotype: w−; +; UAS-OR(w+)/+. To express PstrOR in *D. melanogaster* ab3A OSNs, a series of genetic crosses were performed to obtain flies with red eyes and straight wings carrying genotype w−; ΔHalo/ΔHalo; UAS-OR(w+)/DmelOR22-Gal4 for single sensillum recording. The vector *pUAST.attb* and *Drosophila* strains w−; BI/Cyo; TM2/TM6b and w−; ΔHalo/Cyo; TM2/TM6b were kindly provided by Prof. William B. Walker III from the Swedish University of Agricultural Sciences. The *Drosophila* strain w−; ΔHalo/Cyo; DmelOR22-Gal4 preserved by Prof. Xiaoguang Chen from Southern Medical University was kindly provided by Prof. John R. Carlson from Yale University. 

### 4.7. Single Sensillum Recording

The protocol of single sensillum recording was described in detail previously [51]. A 4–9-day-old fly was wedged into a 200-μL pipette tip. Then the pipette tip close to the head was horizontally cut and the fly head was protruded. The pipette tip was placed on dental wax on a microscope slide with the fly facing upward. The antennae were placed gently on a coverslip and fixed with a glass capillary between the second and third antennae segments. The mounted fly was placed under a high magnification (100×) objective. The reference and recording electrodes were inserted into the right eye and ab3 sensillum, respectively. The electrical signal was amplified using a high impedance DC amplifier (Syntech, Kirchzarten, Germany), digitized through IDAC-4 (Syntech, Kirchzarten, Germany), then recorded and visualized using AutoSpike v3.1 software (Syntech, Kirchzarten, Germany). Signals were recorded for 10 s, starting 1 s before stimulation, and action potentials were counted offline in a 0.5 s period before and after stimulation. Responses were counted by subtracting the spontaneous firing rates 0.5 s before stimulation from the total spike rates 0.5 s after stimulation. Values were recorded in units of spikes/s.

Liquid or solid compounds were diluted in paraffin oil or dimethyl sulfoxide, respectively, to generate a stock solution with a concentration of 10 mg/mL. The concentration gradients of each compound ranging from 10^−5^ to 10 mg/mL were diluted from stock solutions for dose-dependent analysis. To test the response to compounds, a 10-μL portion was applied on a filter strip (0.2 × 30 mm), which was inserted into a Pasteur pipette to build a stimulus cartridge. The antenna was bathed in a high humidity air stream flowing (20 mL/s) from a glass tube. Odor stimulations were delivered to the sensillum through the tip of stimulus cartridge, which was inserted into a hole at the end of the glass tube, and an air pulse (0.5 L/min) lasting 0.5 s was generated using a stimulus controller (CS55, Syntech, Kirchzarten, Germany). Pipettes with filter papers containing the solvent alone served as negative control.

### 4.8. Real-Time Quantitative PCR 

RT-qPCR was performed on the QuantStudio3 Real-Time PCR System (Applied Biosystems, Foster City, CA, USA) using TB Green^®^ Premix Ex Taq^™^ II (Tli RNaseH Plus) (TaKaRa, Dalian, China). *Actin* was used as a reference gene [36]. Primers listed in Table S2 were designed using Beacon Designer 8.0 (Premier Biosoft, Palo Alto, CA, USA). The RT-qPCR reaction mixture included 10 µL of TB Green Premix Ex Taq II (Tli RNaseH Plus) (2×); 0.8 µL each of forward and reverse primer; 0.4 µL of ROX Reference II; 2 μL of cDNA template; and 6 μL of RNase-free water to a final volume of 20 μL. The RT-qPCR program was as follows: 95 °C for 30 s, 40 cycles of 95 °C for 5 s, and 60 °C for 34 s. Three independent biological replicates were tested with three technical replicates for each tissue. Primers for both *actin* and *PstrOR17* individually generated one single peak during amplification, furthermore, the primer efficiencies for both genes were comparable. The relative expression level of *PstrOR17* was calculated using the 2^−∆∆Ct^ method [52].

### 4.9. Sequence and Phylogenetic Analysis

The OR sequences of *P. striolata* used in this study were obtained from a previous study [36]. The OR amino acid sequences were aligned using MAFFT v.7 with the auto-option strategy [53]. The maximum-likelihood tree was generated with IQ-TREE, which runs automatic model testing and selection via the Model Finder function [54]. Before tree construction, LG + F + R6 was selected as the best-fit model according to the Bayesian information criterion. Bootstrap support values were based on 1000 replicates and rooted with the Orco lineage. The tree was visualized and color coded using iTOL (https://itol.embl.de) (accessed on 21 January 2024). 

### 4.10. Homology Modeling and Molecular Docking

The three-dimensional (3D) structure of PstrOR17 was modeled using AlphaFold2 (https://alphafold.ebi.ac.uk/) (accessed on 27 October 2023) [48]. The quality and rationality of the PstrOR17 model were evaluated online using a PROCHECK Ramachandran plot in SAVES 6.0 (https://saves.mbi.ucla.edu) (accessed on 27 October 2023). The 3D structures of (−)-verbenone and (S)-cis-verbenol were obtained from the PubChem database (https://pubchem.ncbi.nlm.nih.gov) (accessed on 27 October 2023). AutoDock Tools 1.5.7 (ADT) was employed to convert the receptor protein structure and ligand molecular structure into a PDBQT file, and molecular docking was performed using AutoDock Vina 1.1.2. The docking parameters correspond to interaction energies (kcal/mol). The interactions between protein and ligands were analyzed and visualized using PyMol 2.5 and Protein–Ligand Interaction Profiler.

### 4.11. RNA Interference

Template DNA fragments for the synthesis of dsRNA were amplified using specific forward primers containing T7 promoter and respective reverse primers (Table S2). The TranscriptAid™ T7 High Yield Transcription Kit (Ambion, Austin, TX, USA) was used to synthesize the dsRNAs according to the manufacturer’s protocol. The dsRNA of enhanced green fluorescent protein (EFGP) was used as a control.

For injection, beetles were anesthetized with CO_2_ and lined up on the side for injection. Then, 100 ng of each dsRNA (2 μg/μL) was injected into the abdomen of each *P. striolata* adult using Nanoject II TM injector (Drummond Scientific Company, Broomall, PA, USA) under a dissecting microscope (Leica, Wetzlar, Germany). After injection, insects were reared on pakchoi (*Brassica chinensis* L.) under standard conditions. On days 2, 3, 4, and 5 post-dsRNA injection, RT-qPCR was performed to check the efficiency of RNAi. Behavioral assays were performed after confirmation of gene knockdown to confirm the role of OR in the detection of compounds.

### 4.12. Statistical Analysis

Statistical analyses were performed using SPSS Statistics software 26.0 (IBM Inc., Chicago, IL, USA). Figures were generated using GraphPad Prism version 9.4.0 (GraphPad Software, San Diego, CA, USA). For behavioral assays, χ^2^ goodness-of-fit tests were used to assess the preferences of *P. striolata* adults between controls and treatments. Relative EAG responses were analyzed using one-way analysis of variance (ANOVA) followed by Duncan’s multiple comparisons. The Student’s t-test was used to analyze the expression level of *PstrOR17*. 

## 5. Conclusions

In summary, this study aimed to explore the behavior effect and molecular mechanism of (−)-verbenone and (S)-cis-verbenol in *P. striolata*. We found both odorants exhibited a high attraction effect against *P. striolata*, and successfully identified that the antenna-biased PstrOR17 specifically responded to them. However, whether other PstrORs play a role in detecting these odorants is still unknown and needs to be determined. Through RNAi-mediated knockdown assays, we demonstrated the essential role of PstrOR17 in mediating the recognition of (−)-verbenone and (S)-cis-verbenol. Our work extends the knowledge of Coleoptera ORs and delineates the molecular mechanisms underlying olfactory detection in an important Coleoptera pest. Furthermore, the results of this research highlight the potential of (−)-verbenone and (S)-cis-verbenol as attractants for *P. striolata* adults, providing new perspectives for the development of more effective and reliable biological control strategies. 

## Data Availability

Data are contained within the article and Supplementary Materials.

## Supplementary Materials

The following supporting information can be downloaded at https://www.mdpi.com/article/10.3390/ijms25084362/s1.
