# Supplementary material for: Antenna-Biased Odorant Receptor PstrOR17 Mediates Attraction of Phyllotreta striolata to (S)-Cis-Verbenol and (−)-Verbenone
Source: Int J Mol Sci. 2024 Apr 15;25(8):4362. doi: 10.3390/ijms25084362 (PMC11049977; doi:10.3390/ijms25084362)
Supplement: Supplementary file 1 [file ijms-25-04362-s001.zip › ijms-2939403-supplementary.pdf]

Table S1. Compounds used in this study.

Chemical name	CAS	Chemical name	CAS
β-Citronellol	106-22-9	2-Pentadecanone	2345-28-0
2-Phenylethanol	60-18-2	Acetophenone	98-86-2
Geraniol	106-24-1	β-Ionone	79-77-6
Nerol	106-25-2	(±)-Camphor	76-22-2
1-Hexanol	111-27-3	2-Heptanone	110-43-0
1-Heptanol	111-70-6	β-Caryophyllene	87-44-5
1-Nonanol	143-08-8	(−)-Caryophyllene oxide	1139-30-6
(S)-cis-Verbenol	18881-04-4	Myrcene	123-35-3
Farnesol	4602-84-0	Limonene	138-86-3
(−)-Borneol	464-45-9	(−)-β-Pinene	18172-67-3
Nerolidol	7212-44-4	(E)-β-Farnesene	18794-84-8
(+)-Cedrol	77-53-2	Camphene	79-92-5
3,7-Dimethyl-3-octanol	78-69-3	α-Pinene	80-56-8
Linalool	78-70-6	(−)-trans-Caryophyllene	87-44-5
(E)-2-Hexen-1-ol	928-95-0	Cumene	98-82-8
cis-3-Hexen-1-ol	928-96-1	γ-Terpinene	99-85-4
trans-3-Hexen-1-ol	928-97-2	α-Terpinene	99-86-5
(±)-α-Terpineol	98-55-5	Hexane	110-54-3
1-Pentanol	71-41-0	Nonane	111-84-2
4-Penten-1-ol	821-09-0	Decane	124-18-5
cis-2-Penten-1-ol	1576-95-0	Tridecane	629-50-5
Benzaldehyde	100-52-7	Tetradecane	629-59-4
Heptanal	111-71-7	1,4-Diethylbenzene	105-05-5
Phenylacetaldehyde	122-78-1	2,6-Di-tert-butylphenol	128-39-2
Octanal	124-13-0	1,8-Cineole	470-82-6
1-Nonanal	124-19-6	(−)-Verbenone	1196-01-6
(1R)-(−)-Myrtenal	18486-69-6	Indole	120-72-9
3-Vinylbenzaldehyde	19955-99-8	3-Indoleacetonitrile	771-51-7
4-Ethylbenzaldehyde	4748-78-1	3-Indolemethanol	700-06-1

Citral	5392-40-5	Dimethyl Disulfide	624-92-0
Hexanal	66-25-1	Dimethyl Trisulfide	3658-80-8
trans-2-Hexen-1-al	6728-26-3	Methyl ITC	556-61-6
Methyl phenylacetate	101-41-7	Ethyl ITC	542-85-8
Isobutyl phenylacetate	102-13-6	Propyl ITC	628-30-8
Diethyl malonate	105-53-3	Butyl ITC	592-82-5
Ethyl butyrate	105-54-4	Allyl ITC	57-06-7
Octyl acetate	112-14-1	4-Pentenyl ITC	18060-79-2
Methyl salicylate	119-36-8	Phenyl ITC	103-72-0
Ethyl hexanoate	123-66-0	Benzyl ITC	622-78-6
Benzyl acetate	140-11-4	2-Phenylethyl ITC	2257-09-2
Neryl acetate	141-12-8	4-Methoxyphenyl ITC	2284-20-0
Hexyl acetate	142-92-7	3-(Methylthio)propyl ITC）	505-79-3
Nonyl acetate	143-13-5	sec-Butyl ITC	4426-79-3
trans-2-Hexenyl acetate	2497-18-9	3-Buten-1-yl ITC	3386-97-8
cis-3-Hexenyl acetate	3681-71-8	iso-Butyl ITC	591-82-2
(±)-α-Terpinyl acetate	80-26-2	3-Methoxyphenyl ITC	3125-64-2
Methyl benzoate	93-58-3	Sulforaphane	4478-93-7
4′-Ethylacetophenone	937-30-4	Sulforaphene	592-95-0

Table S2 Primers used in this study.

Primer name	Sequence (5′–3′)
For amplification	
PstrOR9-F	ATGGGGGAAATGCGCGATATAAA
PstrOR9-R	TTATTTATTCATGCTTATGAGTA
PstrOR11-F	ATGACGTCCGAAGGGATTGTGCC
PstrOR11-R	CTTTAGTACATTCATTCTTTTAA
PstrOR17-F	ATGAAACACCTTCAGATAGCCA
PstrOR17-R	CTCAATCGCCTCTTTTGTTATTC
PstrOR26-F	ATGGACTTGGTTTATCCGCAACC
PstrOR26-R	CTAGTTTTCCAGGTGGTTTCTAA
PstrOR38-F	ATGTTCCTGCGAGATATTTTTCC
PstrOR38-R	TCACTTTTCATTATTCACATTGT
PstrOR59-F	ATGTCTTCACCCGATAGACCCAG
PstrOR59-R	TGATGTCTCTAAACGAACAATAA
For RT-qPCR analysis	
PstrOR17-qF[a]	GCCATATCGCAATCAGTT
PstrOR17-qR[a]	CTCGCATCATTACAATCAATAG
actin-qF	TGTCCCACACTGTACCCATC
actin-qR	CGTGGCCATTTCCTGTTCAA
For dsRNA synthesis	
dsPstrOR17-F	TAATACGACTCACTATAGGGCAACATCCAACTGATCGACG
dsPstrOR17-R	TAATACGACTCACTATAGGGTTCGAATTTGACCTGCAAGA

[a] Primers referred to by Wu et al.[1]

T7 promoter sequence in the RNAi primers is underlined.

1. Wu, Z.Z.; Bin, S.Y.; He, H.L.; Wang, Z.B. Differential expression analysis of chemoreception genes in the striped slea beetle *Phyllotreta striolata* using a transcriptomic approach. *Plos One* **2016,** *11*, e0153067.

Figure S1. Ramachandran plot and statistics of PstrOR17 model analyzed by PROCHECK.

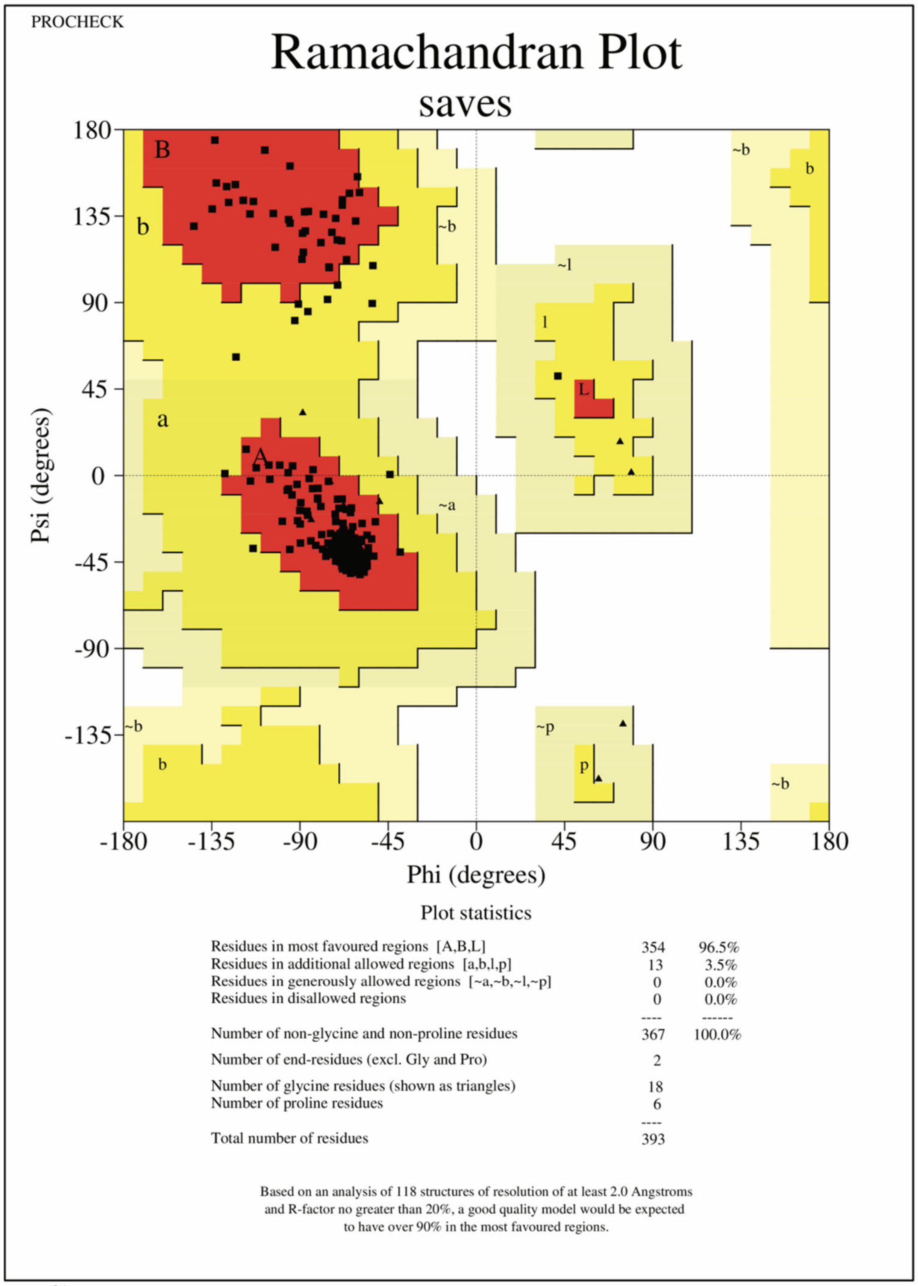

saves_01.ps
